# Xanthurenic Acid Activates mGlu2/3 Metabotropic Glutamate Receptors and is a Potential Trait Marker for Schizophrenia

**DOI:** 10.1038/srep17799

**Published:** 2015-12-08

**Authors:** Francesco Fazio, Luana Lionetto, Martina Curto, Luisa Iacovelli, Michele Cavallari, Cristina Zappulla, Martina Ulivieri, Flavia Napoletano, Matilde Capi, Valentina Corigliano, Sergio Scaccianoce, Alessandra Caruso, Jessica Miele, Antonio De Fusco, Luisa Di Menna, Anna Comparelli, Antonella De Carolis, Roberto Gradini, Robert Nisticò, Antonio De Blasi, Paolo Girardi, Valeria Bruno, Giuseppe Battaglia, Ferdinando Nicoletti, Maurizio Simmaco

**Affiliations:** 1 I.R.C.C.S. Neuromed, Pozzilli, Italy; 2 Advanced Molecular Diagnostics Unit, Sant’Andrea Hospital, Rome, Italy; 3 School of Medicine and Psychology NESMOS Department, Sant’Andrea Hospital, Sapienza University, Rome, Italy; 4 Department of Physiology and Pharmacology, Sapienza University, Rome, Italy; 5 Department of Experimental Medicine, Sapienza University, Rome, Italy; 6 Center for Neurological Imaging, Brigham and Women’s Hospital, Harvard Medical School, Boston, MA, U.S.A; 7 I.R.C.C.S. Fondazione Santa Lucia, Rome, Italy; 8 Department of Molecular Medicine, Sapienza University, Rome, Italy

## Abstract

The kynurenine pathway of tryptophan metabolism has been implicated in the pathophysiology of psychiatric disorders, including schizophrenia. We report here that the kynurenine metabolite, xanturenic acid (XA), interacts with, and activates mGlu2 and mGlu3 metabotropic glutamate receptors in heterologous expression systems. However, the molecular nature of this interaction is unknown, and our data cannot exclude that XA acts primarily on other targets, such as the vesicular glutamate transporter, in the CNS. Systemic administration of XA in mice produced antipsychotic-like effects in the MK-801-induced model of hyperactivity. This effect required the presence of mGlu2 receptors and was abrogated by the preferential mGlu2/3 receptor antagonist, LY341495. Because the mGlu2 receptor is a potential drug target in the treatment of schizophrenia, we decided to measure serum levels of XA and other kynurenine metabolites in patients affected by schizophrenia. Serum XA levels were largely reduced in a large cohort of patients affected by schizophrenia, and, in patients with first-episode schizophrenia, levels remained low after 12 months of antipsychotic medication. As opposed to other kynurenine metabolites, XA levels were also significantly reduced in first-degree relatives of patients affected by schizophrenia. We suggest that lowered serum XA levels might represent a novel trait marker for schizophrenia.

The kynurenine pathway of tryptophan metabolism generates neuroactive compounds that are able to interact with glutamate receptors in the CNS^1^. The first step of the pathway is the conversion of tryptophan into N-formylkynurenine catalyzed by either indolamine 2,3-dioxygenase (IDO) or tryptophan 2,3-dioxygenase (TDO). N-formylkynurenine is converted by formamidase into kynurenine (KYN), which is hydroxylated into 3-hydroxykynurenine (3-HK) by kynurenine monooxygenase (KMO), or, alternatively, transaminated into kynurenic acid (KYNA) by types 1 and 2 kynurenine aminotransferases (KATs), or metabolized into anthranylic acid (ANA) by kynureninase. 3-HK is sequentially transformed into 3-hydroxyanthranylic acid (3-HANA) and quinolinic acid (QUINA)^1^ (Fig. 1). Because brain levels of IDO and TDO are relatively low, KYN and 3-HK of peripheral origin are considered as main sources for brain KYNA, QUINA, and other metabolites of the kynurenine pathway^1^. Therefore, peripheral KYN and 3-HK are reliable indicators of the activity of the kynurenine pathway in the CNS.

KYNA and QUINA have been the subject of extensive investigation since they have been shown to interact with ionotropic glutamate receptors. QUINA acts as an orthosteric agonist at the GluN2 subunits of N-methyl-D-aspartate (NMDA) receptors^2^,^3^. In contrast, KYNA acts as a competitive antagonist at the glycine site of the GluN1 subunit of NMDA receptors, thereby inhibiting NMDA receptor function^4^.

KMO activity is reduced in the brain of patients affected by schizophrenia, resulting in an increased formation of KYNA at the expenses of 3-HK and its downstream metabolites, including QUINA^5^,^6^,^7^,^8^. The imbalance between KYNA and QUINA supports the glutamatergic hypothesis of schizophrenia, which is based on a hypofunction of NMDA receptors in cortical GABAergic interneurons^9^,^10^,^11^,^12^.

Recently, two additional metabolites of the kynurenine pathway, cinnabarinic acid and xanthurenic acid (XA) have been suggested to interact with glutamate receptors. Cinnabarinic acid is formed spontaneously by the condensation of two molecules of 3-HANA, whereas XA is the transamination product of 3-HK. Similar to KYNA, brain neosynthesis of XA is catalyzed by KAT-2 in non-neuronal cells^13^. Cinnabarinic acid behaves as a weak orthosteric agonist of type-4 metabotropic glutamate receptors (mGlu4 receptors)^14^. XA mimicked the action of the mGlu2/3 receptor agonist, LY354740, in reducing the inhibition of ventrobasal thalamic nuclei by the thalamic reticular nucleus upon physiological sensory stimulation. This action of XA was abrogated by the preferential mGlu2/3 receptor antagonist, LY341495, but was not amplified by the selective mGlu2 receptor enhancer, LY487379^15^. XA also mimicked the action of LY354740 in depressing excitatory synaptic transmission in the hippocampal dentate gyrus and CA1 region. However, in these regions the action of XA was insensitive to LY341495^16^. Thus, whether or not XA is able to activate mGlu2/3 receptors is uncertain.

mGlu2 receptors are considered as drug targets in the treatment of schizophrenia because they negatively regulate the activity of type-2A serotonin receptors (5-HT_2A_ receptors), thereby restraining the excitatory thalamic input to cortical pyramidal neurons^17^,^18^. The kynurenine pathway generates a number of neuroactive metabolites that might be directly implicated in the pathophysiology of schizophrenia, of which QUINA and XA lie downstream of KMO, whereas KYNA is a direct metabolic product of KYN. A possible link between XA and schizophrenia has been suggested more than 50 years ago^19^,^20^. Since then, no further studies have been performed on XA and schizophrenia at preclinical and clinical levels. Here, we have further explored the interaction between XA and mGlu2/3 receptors using *in vitro* and *in vivo* models, and we assessed serum levels of XA and other kynurenines in a large cohort of patients affected by schizophrenia, their first-degree relatives, and healthy controls.

## Results

### Study of the interaction between XA and mGlu2/3 receptors

#### XA activated mGlu2 and mGlu3 receptor signaling in heterologous expression systems

We applied XA to HEK293 cells expressing mGlu2, mGlu3, mGlu4, or mGlu7 receptors. XA reduced forskolin-stimulated cAMP formation in cells expressing mGlu2 or mGlu3 receptors in a concentration-dependent fashion. XA potency was apparently greater in mGlu3-expressing cells, in which concentrations as low as 1 nM significantly reduced forskolin-stimulated cAMP formation (Fig. 2A,B). In another experiment we compared the action of XA (30 μM) with that of the prototypical mGlu2/3 receptor agonist, 2R,4R-APDC (100 μM). The two compounds were equally efficacious in inhibiting forskolin-stimulated cAMP formation in both mGlu2- and mGlu3-expressing cells, and their action was abrogated by the preferential mGlu2/3 receptor antagonist, LY341495 (Fig. 2A,B). Remarkably, XA failed to affect forskolin-stimulated cAMP formation in cells expressing mGlu4 and mGlu7 receptors, as well as in mock cells (Fig. 2C–E). mGlu4- and mGlu7-expressing cells responded to the group-III mGlu receptor agonist, L-2-amino-4-phosphonobutanoate (L-AP4) (Fig. 2C,D), as expected^21^ (Fig. 2C,D). XA (30 or 300 μM) had no effect on cAMP formation in the absence of forskolin in mock cells or in cells expressing mGlu2, −3, −4, or −7 receptors (not shown).

#### Effect of XA on signal transduction mechanisms of native mGlu2/3 receptors

**Figure 1:**
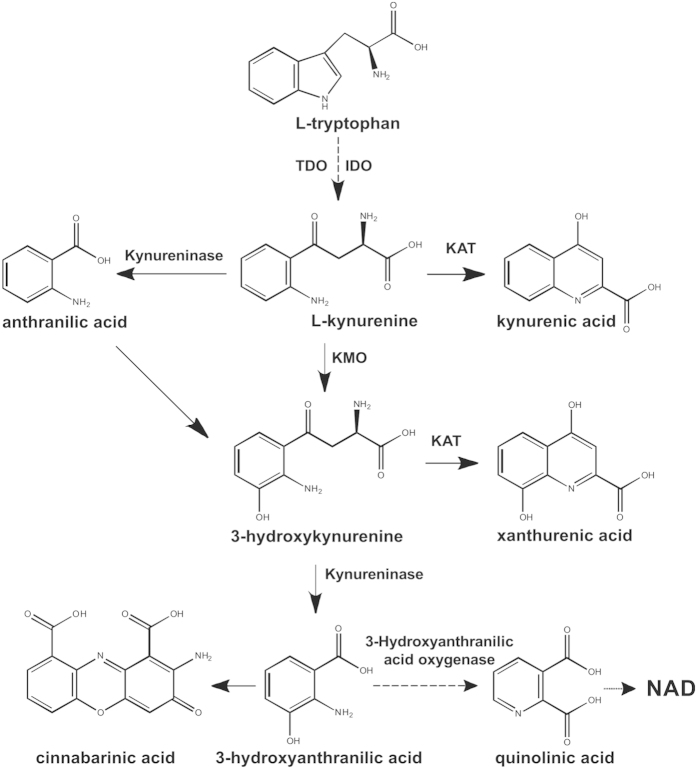
The kynurenine pathway of tryptophan. TDO = tryptophan 2,3-dioxygenase; IDO = indoleamine 2,3-dioxygenase; KAT = kynurenine aminotransferase; KMO = kynurenine 3-monooxygenase; NAD = nicotinamide adeninedinucleotide.

**Figure 2:**
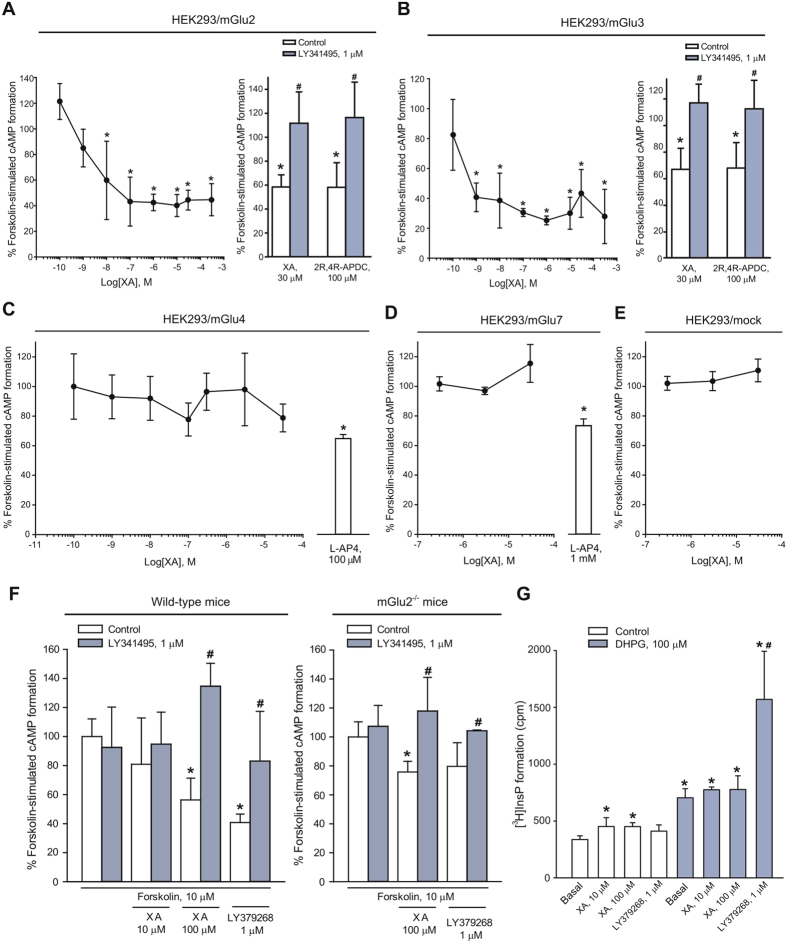
Xanthurenic acid (XA) activates mGlu2/3 receptor signaling in heterologous expression systems and brain tissue. (**A**) Left: concentration-dependent inhibition of forskolin-stimulated cAMP formation by XA in HEK293 cells expressing mGlu2 receptors. Values are means + S.D. of 2–5 determinations. *p < 0.05 vs. forskolin alone (One-way ANOVA + Fisher’s LSD; F_(8,24)_ = 5.8). Right: inhibition of forskolin-stimulated cAMP formation by 2R,4R-APDC or XA in the absence or presence of LY341495. Values (means + S.E.M.; n = 4) were extrapolated from a different experiment with additional groups not shown here. p < 0.05 vs. forskolin alone (*) or vs. forskolin + 2R,4R-APDC or forskolin + XA (#) (One-way ANOVA + Fisher’s LSD; F_(11,36)_ = 22.326). (**B**) Left and Right: same as in (**A**), but in mGlu3-expressing HEK293 cells. Left: values are means + S.D. of 2–3 determinations. *p < 0.05 vs. forskolin alone (One-way ANOVA + Fisher’s LSD; F_(8,25)_ = 10.49). Right: values (means + S.D.; n = 4) were extrapolated from a different experiment with additional groups not shown here. p < 0.05 vs. forskolin alone (*) or vs. forskolin + 2R,4R-APDC or forskolin + XA (#) (One-way ANOVA + Fisher’s LSD; F_(11,36)_ = 26.913). XA (30 or 300 μM) had no effect on cAMP in the absence of forskolin in both (**A,B**). (**C–E**) XA fails to inhibit forskolin-stimulated cAMP formation in mGlu4- and mGlu7-expressing cells or in mock cells. Values are means + S.D. of 4 determinations. *p < 0.05 vs. forskolin alone (One-way ANOVA + Fisher’s LSD; (**C**) F_(8,26)_ = 18.36; (**D**) F_(4,19)_ = 12.03. (**F**) Inhibition of cAMP formation by XA or LY379268 in cortical slices prepared from wild-type and mGlu2^−/−^ mice. Values are means + S.D. of 4 determinations; p < 0.05 vs. the respective control values (*) or vs. the respective values obtained in the absence of LY341495 (#). (One-way ANOVA + Fisher’s LSD; wild-type mice: F_(7,21)_ = 4.885; mGlu2^−/−^ mice: F_(5,14)_ = 4.478. (**G**) Stimulation of PI hydrolysis in cortical slices incubated in the absence or presence of DHPG, XA, or LY379268. Values are means + S.D. of 4–5 determinations; p < 0.05 vs. basal values of the control group (*) or the DHPG group (#) (One-way ANOVA + Fisher’s LSD; F_(7,25)_ = 24.663.

We examined the interaction between XA and native mGlu2/3 receptors using rat cortical slices. In this preparation, XA (100 μM) mimicked the action of the mGlu2/3 receptor agonist, LY379268 (1 μM), in inhibiting forskolin-stimulated cAMP formation, and its action was antagonized by LY341495 (1 μM) (Fig. 2F). In cortical slices prepared from mGlu2^−/−^ mice, XA was still able to reduce forskolin-stimulated cAMP formation, but to a lower extent than in slices from wild-type mice (−44% and −25% in wild-type and mGlu2^−/−^ mice, respectively). The residual activity of XA in slices from mGlu2^−/−^ mice was antagonized by LY341495 (Fig. 1F). Of note, at least under our experimental conditions, L-AP4 (1 or 100 μM) did not inhibit forskolin-stimulated cAMP formation in cortical slices (not shown).

We extended the analysis to another signal transduction mechanism activated by native mGlu2/3 receptors, i.e. the amplification of mGlu1/5-receptor mediated PI hydrolysis in brain slices^22^,^23^. As expected, the mGlu2/3 agonist, LY379268, had no effect on [^3^H]inositolmonophosphate (InsP) formation *per se*, but amplified the stimulation of [^3^H]InsP formation produced by the mGlu1/5 receptor agonist, DHPG, in adult mouse cortical slices. In contrast, XA (10 and 100 μM) caused a small increase in [^3^H]InsP formation on its own, but failed to amplify the action of DHPG (Fig. 2G).

#### *Analysis of [* ^*3*^ *H]XA binding in HEK 293 cells and cortical membranes*

**Figure 3:**
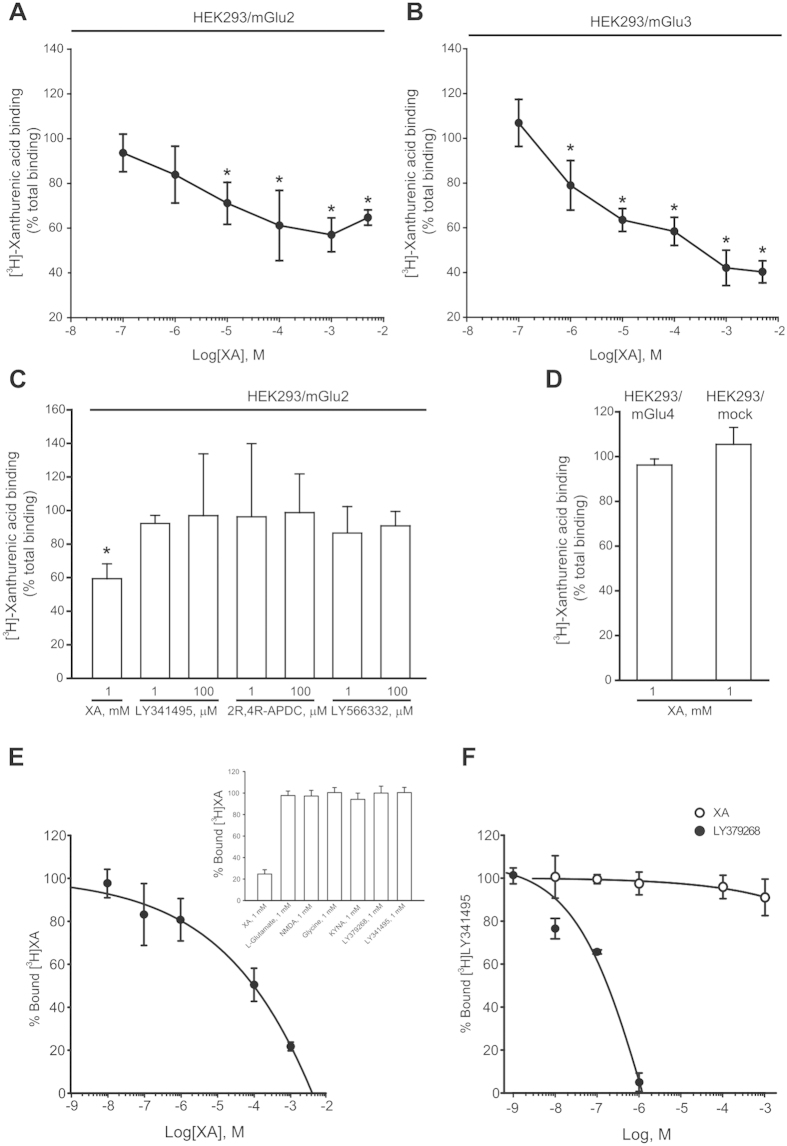
[^3^H]XA binding in membranes prepared from transfected HEK293 cells or mouse cortical membranes. (**A,B**) Binding of [^3^H]XA (5 nM) in membranes prepared from HEK293 cells expressing mGlu2 or mGlu3 receptors. Values are means + S.D. of 2–3 determinations. *p < 0.05 (One-way ANOVA + Fisher’s LSD; F values: (**A**) F_(6,19)_ = 5.73; (**B**) F_(6,20)_ = 23.24. (**C**) Binding of [^3^H]XA binding (5 nM) in membrane prepared from HEK293 cells expressing mGlu2 receptors incubated in the presence of orthosteric or allosteric mGlu2 receptor ligands. Values are means + S.D. of 3–6 determinations. *p < 0.05 (One-way ANOVA + Fisher’s LSD; F_(7,32)_ = 2.56). (**D**) Excessive concentrations of XA (1 mM) fail to inhibit [^3^H]XA binding in membranes from HEK293 cells expressing mGlu4 receptors, or in membranes from mock cells. Values are means + S.D. of 3–6 determinations. (**E**) Concentration-dependent inhibition of [^3^H]XA (5 nM) binding by non-radioactive XA in mouse cortical membranes. The lack of effect of mGlu and NMDA receptor ligands on [^3^H]XA binding is shown in the inset. (**F**) [^3^H]LY341495 (1 nM) binding in mouse cortical membranes incubated in the presence of increasing concentrations of XA or LY379268. In (**E,F**), values are means ± S.D. of triplicates.

[^3^H]XA is known to bind to specific and saturable recognition sites in brain membranes^24^,^25^. We specifically examined whether [^3^H]XA binding required the presence of mGlu2 or mGlu3 receptors, and whether specifically bound [^3^H]XA could be displaced by orthosteric or allosteric ligands of these receptors. In membranes prepared from mGlu2- or mGlu3-expressing HEK293 cells, non-radioactive XA inhibited [^3^H]XA binding in a concentration-dependent fashion, with an apparent IC_50_ value of 10 μM (both cell types), and maximal inhibition of about 40% and 60% in mGlu2- and mGlu3-expressing membranes, respectively. Using membranes from mGlu2-expressing cells, we examined whether two orthosteric mGlu2/3 receptor ligands (LY341495, 2R,4R-APDC), and one selective allosteric ligand of mGlu2 receptors (LY566332) could displace specifically bound [^3^H]XA. None of these drugs (tested at concentrations of 1 or 100 μM) significantly inhibited [^3^H]XA binding (Fig. 3C). Interestingly, high concentrations of non-radioactive XA (1 mM) did not inhibit [^3^H]XA binding in membranes prepared from mock cells or mGlu4-expressing cells, suggesting that specific [^3^H]XA binding in HEK293 cells requires the expression of either mGlu2 or mGlu3 receptors (Fig. 3D).

In crude synaptic membranes prepared from the mouse cerebral cortex, [^3^H]XA binding was inhibited by non-radioactive XA with an apparent IC_50_ value of about 10 μM (Fig. 3E). Specifically bound [^3^H]XA was not displaced by L-glutamate, LY379268, and LY341495. Binding was also unaffected by NMDA, glycine, and KYNA (see inset of Fig. 3E). Using cortical membranes, we also examined whether XA could interact with the recognition site of the mGlu2/3 receptor antagonist, LY341495. [^3^H]LY341495 binding was inhibited by non-radioactive LY379268 in a concentration range from 10 nM to 1 μM, as expected (Fig. 3F). In contrast XA (100 nM–1 mM) failed to inhibit [^3^H]LY341495 in cortical membranes (Fig. 3F).

#### Exogenous XA displayed antipsychotic-like activity requiring the presence of mGlu2 receptors

**Figure 4:**
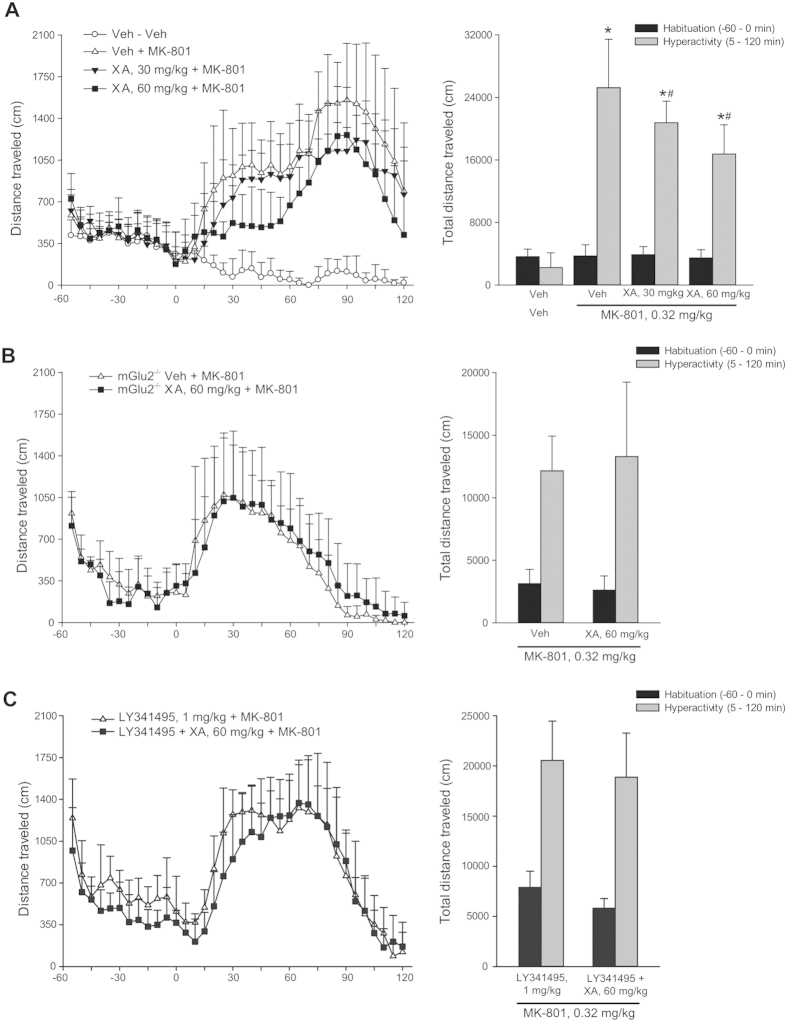
Xanthurenic acid (XA) displays antipsychotic-like activity in mice challenged with MK-801. (**A**) Locomotor activity in mice pre-treated with saline (vehicle) or two doses of XA (30 or 60 mg/kg, i.p.) for 60 min, and then challenged with MK-801. Locomotor activity was expressed both as a function of time in 5 min beans (left) and as the sum of the total travelled distance in the habituation and hyperactivity phases (right). Values are means + S.D. of 6–8 determinations. p < 0.05 (One-way ANOVA + Fisher’s LSD) vs. values of the hyperactivity phase obtained in mice that did not receiveMK-801 (i.e., mice treated with veh + veh) (*), or vs. values obtained in mice treated with MK-801 without pre-treatment with XA (veh + MK-801); F_(3,25)_ = 36.142. Veh = vehicle. (**B**) Same as in A) in mGlu2^−/−^ mice. Here, only the dose of 60 mg/kg of XA was tested. Values are means + S.D. of 6–8 determnations. (**C**) Same as in A), but in normal mice treated i.p. with LY341495 (1 mg/kg) combined with either vehicle or XA (60 mg/kg) for 60 min and challenged with MK-801.

Mice were injected i.p. with saline or two doses of XA (30 or 60 mg/kg) 60 min prior to MK-801 (0.32 mg/kg. i.p.), and examined for an overall period of 180 minutes (60 minutes before, and 120 after, MK-801 treatment). In mice pretreated with saline, MK-801 caused a sharp increase in locomotor activity, which showed a biphasic kinetic with a first plateau phase from 30 to 75 min, and a subsequent peak at 85–110 min; hyperactivity was still prominent at the end of the observation period (Fig. 4A). XA had no effect on locomotor activity during the 60-min habituation phase, but significantly reduced MK-801-induced hyperactivity in a dose-dependent manner (Fig. 4A).

A similar experimental protocol was applied to wild-type and mGlu2^−/−^ mice. In wild-type mice, we confirmed that 60 mg/kg of XA was able to reduce MK-801-induced hyperactivity (not shown). In contrast, XA was inactive in mGlu2^−/−^ mice (Fig. 4B). Of note, however, mGlu2^−/−^ mice showed a lower motor response to MK-801, which could have occluded the effect of XA. We therefore performed an additional experiment in which normal mice were challenged by XA combined with the preferential mGlu2/3 receptor antagonist, LY341495 (1 mg/kg). XA-failed to reduce MK-801-induced hyperactivity in the presence of LY341495 (Fig. 4C). Taken collectively, these findings suggest that the antipsychotic-like activity of XA required the activation of mGlu2 receptors.

#### Measurements of XA and other kynurenine metabolites in the serum of patients affected by schizophrenia, their first-degree relatives, and healthy controls

Demographic and clinical features of patients affected by schizophrenia (n = 90), first-degree relatives (n = 25), and healthy controls (n = 84) are shown in Tables 1, 2, 3. Patients affected by schizophrenia showed a prevalence of male gender, higher body mass index (BMI), higher percentage of subjects with a history of drug and alcohol abuse, and higher percentage of current cigarette smokers with respect to healthy controls or first-degree relatives. First-degree relatives of patients showed higher BMI and PANSS (positive and negative symptoms scale) scores with respect to healthy controls (Table 1).

Patients affected by schizophrenia were separated into FES (“first-episode schizophrenia”) and MES (“multi-episode schizophrenia”). The two subgroups showed different demographic and clinical characteristics, with FES patients showing lower BMI and a higher percentage of past alcohol abusers. GAF (Global Assessment of Function) scores were similar in the two groups. Scores of PANSS subscales were significantly different between FES and MES patients, with positive symptoms being more prevalent in FES patients, as expected (Table 2). Fourteen FES patients were re-examined after 12 months of treatment with atypical antipsychotic drugs. This treatment reduced PANSS scores of general and positive symptoms, and also improved CGI (Clinical Global Impression) and GAF scores (Table 3).

#### Changes in the levels of XA and other kynurenine metabolites in the serum of patients, relatives, and controls

LC/MS-MS analysis of kynurenine metabolites showed large and significant reductions in the levels of 3-HK (−59%), XA (−51%), 3-HANA (−48%), and QUINA (−41%) in the serum of the overall population of patients affected by schizophrenia, as compared to healthy controls (Table 1). Levels of KYNA and ANA were significantly increased in patients (+16% and +65%, respectively), whereas levels of KYN, Trp, and 5-HIAA did not differ (Table 1).

Levels of XA and 3-HANA were also reduced in first-degree relatives of patients with respect to healthy controls (−40% and −27%, respectively); only XA serum levels did not differ between patients and their relatives, but patients had significantly lower 3-HANA levels than relatives. Levels of Trp, KYN and KYNA were higher in relatives of patients than in healthy controls (Table 1).

The subgroup of FES patients also showed a substantial reduction in serum levels of XA (−59%), 3- HK (−83%), 3-HANA (−71%), and QUINA (−41%), and an increase in the levels of ANA (+111%) with respect to healthy controls (Table 2). In MES patients, levels of XA (−48%) and QUINA (−39%) were reduced to a similar extent, whereas changes in the levels of 3-HK (−47%), 3-HANA (−37%), and ANA (+42%) were smaller than in FES patients (Table 2).

One-year treatment with atypical antipsychotics in fourteen FES patients caused large increases in levels of 3-HK (+466%) and 3-HANA (+261%), but no changes in XA or QUINA levels (Table 3).

#### Correlations between serum levels of kynurenine metabolites and clinical scales in patients affected by schizophrenia

In the overall population of patients and in the subgroup of FES patients, there were no significant correlations between GAF, CGI, and PANSS (total or subscale) scores and any of the kynurenine metabolites. Weak correlations were found between KYN, KYNA or QUINA levels and PANSS or CGI scores in MES patients (see Supplementary Information and Table S5). A few correlations were found between kynurenine metabolites (with the exception of XA) and the cognitive domains of the Neuropsychological Test Battery (Table S6-S9). Of note, serum XA levels did not correlate with scores of the clinical scales in any subgroup of patients.

## Discussion

XA is considered as a putative neurotransmitter in the CNS, being stored in synaptic vesicles and released by membrane depolarization in a Ca^2+^-dependent fashion^25^. The demonstration of specific and saturable [^3^H]-XA binding and the evidence that XA stimulates [^35^S]GTP-γ-S binding in brain membranes^24^ suggests that XA interacts with G-protein coupled receptors. Because the electrophysiological effects of XA were blocked by LY341495 in the thalamus, but not in the hippocampus^15^,^16^, it has been suggested that XA does not directly interact with mGlu2 or mGlu3 receptors. XA is known to inhibit vesicular glutamate transporters (VGLUTs)^26^,^27^, and this has been considered as the primary mechanism responsible for the electrophysiological effects of XA in the hippocampus and other brain regions^16^,^28^. Present data do not exclude this possibility, but support the hypothesis that XA may directly activate mGlu2 and mGlu3 receptors. In heterologous expression systems, XA could activate both mGlu2 and mGlu3 receptors with high potency, but had no activity on mGlu4 and mGlu7 receptors. In addition, specific [^3^H]XA binding could be detected in membranes prepared from cells expressing mGlu2 or mGlu3 receptors, but not in membranes prepared from mock cells or from cells expressing mGlu4 or mGlu7 receptors. How precisely XA interacts with mGlu2 and mGlu3 receptors is unclear. It is unlikely that XA binds to the glutamate recognition site of mGlu2 or mGlu3 receptors because [^3^H]XA binding was not affected by orthosteric mGlu2/3 receptor ligands, and XA could not displace specifically bound [^3^H]LY341495^15^. The mGlu2 receptor PAM, LY566332, was also unable to inhibit [^3^H]XA binding, in membranes prepared from mGlu2-expressing cells. This, however, does not exclude that XA binds to an allosteric site that does not overlap with the LY566332 binding site. Alternatively, XA might interact with a different receptor protein that may signal only in the presence of mGlu2 or mGlu3 receptors. Data obtained in brain tissue also suggest, but do not prove, that XA interacts with mGlu2 and mGlu3 receptors. In cortical slices, XA mimicked the action of LY379268 in reducing cAMP formation, but, unlike LY379268, it failed to amplify mGlu1/5 receptor-mediated PI hydrolysis. One possible explanation for these findings is that XA acts as a biased mGlu2/3 agonist^29^ by directing receptor activation towards some, but not all, signalling pathways. An alternative possibility is that VGLUT inhibition by XA may cause a non-vesicular release of glutamate, which in turn activates Gi-coupled group-III mGlu receptors. The concentration of LY341495 that we have used for cAMP experiments in cortical slices (1 μM) might be sufficient to block at least mGlu7 and mGlu8 receptors^21^. However, XA had no effect on mGlu7 receptors in recombinant cells, and, in cortical slices, concentrations of L-AP4 that fully activate mGlu8 receptors (100 μM) did not change forskolin-stimulated cAMP formation under our experimental conditions. Thus, it is unlikely that the action of XA on cAMP formation in cortical slices is mediated by an enhanced release of glutamate and subsequent activation of group-III mGlu receptors.

The evidence that mGlu2 receptors negatively regulate responses mediated by 5-HT_2A_ receptors^17^,^18^ paved the way for the development of mGlu2/3 receptor agonists or mGlu2 receptor positive allosteric modulators (PAMs) in the treatment of schizophrenia. We found that exogenous XA, administered systemically to mice at doses that enhance CNS XA levels^25^, displayed antipsychotic-like activity in mice challenged with MK-801. This effect was abolished in mGlu2^−/−^ mice, in line with the evidence that the antipsychotic-like activity of mGlu2/3 receptor agonists is exclusively mediated by mGlu2 receptors^30^,^31^. This suggests that XA behaves as an endogenous “antipsychotic-like” compound by activating mGlu2 receptors, and that peripheral XA can cross the blood-brain barrier and influence the activity of brain regions that are involved in the pathophysiology of schizophrenia^25^.

We examined blood levels of XA and other kynurenine metabolites in a relatively large cohort of patients affected by schizophrenia, taking into account the following aspects: (i) peripheral KYN and 3-HK enter the brain in large amounts, and fuel the kynurenine pathway in the CNS, whereas brain penetration of KYNA, ANA, 3-HANA, and QUINA, is poor^1^; and (ii) enzymes of the kynurenine pathway are segregated in different cell types in the CNS, with KMO and kynureninase being present in microglia, and KATII in astrocytes^32^,^33^. No segregation exists in the periphery, and this may help to explain why targeted KMO deletion in mice causes a strong reduction in QUINA levels in the plasma and liver, but only a slight reduction in the brain, whereas other kynurenine metabolites showed similar changes in the periphery and in the CNS^34^. Thus, blood levels of at least KYN, 3-HK, and XA might be considered as reliable indicators of the brain kynurenine pathway, whereas this correlation is weaker for QUINA.

Serum levels of kynurenine metabolites that lie downstream of KMO (i.e., 3-HK, XA, 3-HANA, and QUINA) were largely reduced in patients affected by schizophrenia, whereas levels of KYNA and ANA, which originate from KYN *via* KMO-independent reactions, were increased. These changes are consistent with the evidence that KMO activity is reduced and CSF KYNA levels are increased^1^,^35^,^36^,^37^. The strong reduction in XA levels found in both subgroups of schizophrenia patients is consistent with the hypothesis that a reduced activation of mGlu2 receptors is involved in the pathophysiology of schizophrenia.

FES patients represent an ideal population for the search of “glutamatergic” biomarkers without the confounding effects of chronic exposure to antipsychotic medication. A growing body of evidence suggests that a glutamatergic dysfunction already occurs in the prodromal phase of schizophrenia and is not affected by antipsychotic treatment^38^. In FES patients, we found the same changes of kynurenine metabolites observed in the overall population of patients, including a strong reduction in XA levels, even though none of the FES patients was taking antipsychotic medication, suggesting that abnormalities in kynurenine metabolites are neither reduced nor normalized by treatment. However, our findings for 3-HK and KYNA (reduction and increase, respectively) are not in agreement with previous studies showing that plasma 3-HK levels were increased or unchanged^39^,^40^,^41^, whereas KYNA levels were reduced in FES patients^39^. We have no explanation for these contrasting findings.

Interestingly, XA levels were not normalized by one year of treatment with atypical antipsychotic drugs, suggesting that a potential peripheral source of brain mGlu2 receptor activation remains low in spite of the improvement of symptoms in response to medication. This, combined with the evidence that cortical mGlu2 receptors are epigenetically down-regulated by atypical antipsychotics^42^, suggests that a hypofunction of mGlu2 receptors may persist during treatment, making the therapeutic response to antipsychotic drugs sub-optimal. It was unexpected that levels of 3-HK and 3-HANA were increased by antipsychotic treatment, whereas levels of XA and QUINA were not. It is possible that enzymes of the kynurenine pathway other than KMO are altered in schizophrenia and that antipsychotic drugs regulate the pathway at multiple levels.

As in patients, XA levels were substantially reduced in their first-degree relatives and did not correlate with scores on the clinical scales or neuropsychological tests. Even though other Axis II schizophrenia spectrum disorders were not excluded, this suggests that a reduction in XA levels represents a trait marker, but not a state marker, for schizophrenia.

In conclusion, our data demonstrate for the first time that peripheral levels of XA are markedly reduced in patients affected by schizophrenia, regardless of stage of the disorder or medication status. Data obtained in first-degree relatives suggest that peripheral XA levels can be measured as predictive biomarkers in people or family at risk to develop the disorder, but larger studies are needed to verify this hypothesis. In addition, low peripheral XA levels might contribute to a blunted endogenous activation of mGlu2/3 receptors in the CNS, and that subgroup of patients may be more likely to respond to mGlu2/3 receptor agonists or mGlu2 receptor PAMs. Drugs that enhance XA levels might be helpful in the treatment of patients affected by schizophrenia, particularly in the early phases of the disease in which normalization of glutamatergic function represents an unmet therapeutic goal.

## Methods

### Ethics statement

Clinical samples were collected at the Sant’Andrea Hospital, Psychiatry Unit. All serm samples were collected in accordance with the approved guidelines and relevant regulations. All subjects provided their written informed consent prior to participating in the study. The study design and all experimental procedures were approved by the Ethical Committee at Sant’Andrea Hospital, Sapienza University, Rome, Italy (prot.165/2014). All experiments were performed in accordance with the approved guidelines.

All *in vivo* and *in vitro* experiments were carried out in accordance with the European (86/609/EEC) and Italian (DL.116/92) guidelines of animal care. The experimental protocol was approved by the Italian Ministry of Health (D.M. 209/2011-B).

#### Cell culture, transfection and plasmids

mGlu2, mGlu3, mGlu4 and mGlu7 receptor cDNAs were kindly provided by J. Blahos (Academy of Science, Prague, Czech Republic), F. Ferraguti (Innsbruck Medical University, Innsbruck, Austria), and J.P. Pin (Institute du Génomique Fonctionelle, Montpellier, France), respectively.

Human embryonic kidney (HEK) 293 cells were cultured in Dulbecco’s modified Eagle’s medium (DMEM) supplemented with 10% fetal calf serum, 100 U/ml penicillin, and 100 μg/ml streptomycin. Cells were transfected as described previously^43^,^44^,^45^ in 10-mm Falcon dishes using 8 μl of LipofectAMINE2000 in OptiMEM medium (Invitrogen, Carlsbad, CA), and 15 μg of cDNA. Cells used for cAMP assay were co-transfected with 2.5 μg/dish of adenylyl cyclase type V cDNA^46^. Three μg/dish of excitatory amino acid carrier 1 (EAAC1) cDNA were co-transfected in each experiment. After 4 h, the transfection medium was replaced with culture medium and cells were seeded in 48-well plates previously coated with poly-L-lysine (0.01%). Experiments were performed 72 h after transfection and cells were serum starved 16–18 h before. Cell cultures were incubated at 37 °C in in Hanks’ balanced salt solution (pH 7.4), in the presence of 0.5 mM 3-isobutyl-1-methylxanthine and bovine serum albumin (0.3%). Drugs were added 10 min prior to forskolin (10 μM). The incubation was stopped after 10 min by replacing the buffer with ice-cold PCA (0.4 N).

cAMP levels were determined by either radioimmunoassay (PerkinElmer Life and Analytical Sciences, Waltham, MA) or ELISA (Arbor Assay kit, Ann Arbor; MI). Membranes prepared from mGlu2-expressing cells were also used for [^3^H]XA binding experiments (see Supplementary Information).

#### Animals

Six to eight week-old male C57BL/6J mice (17–25 g) purchased from Charles River (Calco, Italy) were used for experiments on cortical slices and for binding studies. mGlu2^−/−^ mice and their wild-type counterparts were kindly provided by Prof. Shigetada Nakanishi (Kyoto University, Japan) and crossed back up to the 17^th^ generation on a C57BL/6J background. These mice were used for behavioural analysis. Mice were housed under standard conditions with a 12 h light/dark cycle and food and water *ad libitum*. *In vivo* studies were in accordance with the National Guidelines for Animal Use (Italian Parliament DL.116/92) and approved by the Italian Ministry of Health (D.M. 209/2011-B).

#### Binding studies and measurements of signal transduction mechanisms activated by mGlu2/3 receptors

[^3^H]XA and [^3^H]LY341495 binding was determined in membrane preparations from HEK293 cells or mouse cerebral cortex. Inhibition of cAMP formation was assessed as described above (see also Supplementary Information). Stimulation of polyphosphoinositide (PI) hydrolysis was assessed in adult mouse cortical slices, as described previously^47^. Methods are detailed in the Supplementary Information.

#### Assessment of MK-801-stimulated locomotor activity

MK-801-induced hyperactivity has been assessed in wild-type and mGlu2^−/−^ mice treated with XA, as detailed in the Supplementary Information.

#### Measurements of serum levels of XA and other kynurenine metabolites in patients affected by schizophrenia, their first-degree relatives, and healthy controls

We developed a liquid chromatography/tandem mass spectrometry (LC/MS-MS) method for the assay of all kynurenine metabolites. The method allowed a reliable detection of KYN, KYNA, ANA, 3-HANA, 3-HK, XA, and QUINA. Levels of tryptophan (Trp) and 5-hydroxyindolacetic acid (5-HIAA) were also detected. Details on sample preparation, reagents, standard solutions, chromatographic conditions, mass spectrometry conditions, and validation parameters are reported in the Supplementary Information (see Tables S1-S3).

The study design was approved by the Ethical Committee at Sant’Andrea Hospital, University Sapienza, Rome, Italy (prot.165/2014). All subjects included in the study signed a free informed consent. M.C., V.C., and A.C. performed the diagnostic assessment at the Psychiatry Unit of Sant’Andrea Hospital. We recruited 60 patients with a history of multi-episode schizophrenia (MES) from the outpatient clinic and 30 patients with first-episode schizophrenia (FES) from the inpatient clinic, all meeting the diagnostic criteria of DSM-IV-TR. Exclusion criteria were: (i) the presence of psychiatric co-morbidities, systemic inflammatory disorders, endocrine disorders, neurological disorders, and mental retardation; (ii) lifetime history of an Axis I disorder (for healthy volunteers and relatives); and (iii) the use of any neurotropic drug or drug of abuse in the last 3 months (except cigarette smoking). In FES patients, the initial diagnosis was confirmed after a 6-month follow-up interview. Fourteen FES patients were re-examined after 12 months of treatment with antipsychotic drugs. Twenty-five first-degree relatives of patients affected by schizophrenia and 84 healthy volunteers also participated to the study. Healthy volunteers were recruited among the hospital personnel and were all unrelated. Only one of the first-degree relatives (i.e. parents or siblings) for each patient, FES and MES, was included in the study. Blood was collected from all subjects for measurements of serum levels of XA and other kynurenine metabolites. The following scales were administered to all participants: PANSS, CGI, and GAF. Neuropsychological assessment was carried out according to the “Measurement and Treatment Research to Improve Cognition in Schizophrenia”^48^. The following domains were examined: speed of processing, sustained attention/vigilance, working memory, verbal learning, visual learning, reasoning/problem-solving, and social cognition (Table S6). The neurocognitive battery was administered after clinical stabilization with a median time from admission of four weeks (range: 17–35 days).

#### Statistical analysis

One-way ANOVA *plus* Fisher’s LSD was applied to the analysis of data obtained in cell cultures, cortical slices, and mice challenged with MK-801.Mann-Whitney, Kruskal-Wallis or Chi-square tests were used for the analysis of the demographic and clinical variables among the groups of patients and control subjects. Bivariate correlations of serum kynurenine metabolites levels with demographic and clinical variables were assessed using the nonparametric Spearman correlation coefficient (rho). The Wilcoxon signed rank test was applied to the analysis of FES patients after 1 year of treatment with antipsychotic drugs. Two-tailed probability of *p* ≤ 0.05 was considered statistically significant. The STATA software version 11 (SAS/STAT User’s Guide, Version 8, SAS Institute, Cary, N.C., 2000) was used for statistical analyses.

## Additional Information

**How to cite this article**: Fazio, F. *et al*. Xanthurenic Acid Activates mGlu2/3 Metabotropic Glutamate Receptors and is a Potential Trait Marker for Schizophrenia. *Sci. Rep*. **5**, 17799; doi: 10.1038/srep17799 (2015).

## Supplementary Material

Supplementary Information

## Figures and Tables

**Table 1:** Serum levels of xanthurenic acid (XA) and other kynurenine metabolites in the overall population of patients affected by schizophrenia, their first-degree relatives, and healthy controls.

	Healthy controls (n = 84)	Patients with schizophrenia (n = 90)	Relatives of patients (n = 25)
Gender (F, %)	47 (55.9)	28 (31.1)^*^	17 (68.0)^#^
Age (years; mean ± SD)	32.8 ± 10.4	33.4 ± 11.7	60.0 ± 9.9^*#^
BMI (mean ± SD)	23.1 ± 4.0	24.6 ± 4.0^*^	23.5 ± 2.4^*^
Antipsychotic treatment (n,%)	–	60 (66.0)	–
Diagnosis (n,%)			
Schizophrenia			
295.9^a^	–	60 (66.6)	–
Brief psychotic disorder			
298.8^a^	–	30 (33.3)	–
Substance Abuse (n,%)	6 (7.1)	27 (30.0)^*^	0 (0.0) ^#^
Alcohol Abuse (n,%)	6 (7.1)	19 (21.1)^*^	0 (0.0) ^#^
Smoking (n,%)	27 (32.1)	63 (70.0)^*^	9 (25.7)^#^
CGI (mean ± SD)	1.0 ± 0.0	5.3 ± 1.0^*^	1.0 ± 0.0^#^
GAF (mean ± SD)	93. 0 ± 2.8	40.8 ± 14.3^*^	90. 6 ± 2.9^#^
PANSS total (mean ± SD)	32.6 ± 3.3	91.6 ± 17.9^*^	41.2 ± 8.0^*#^
PANSS positive (mean ± SD)	7.0 ± 0.0	19.7 ± 7.2^*^	8.6 ± 2.3^*#^
PANSS negative (mean ± SD)	7.0 ± 0.0	25.4 ± 7.3^*^	8.7 ± 3.5^*#^
PANSS general (mean ± SD)	18.6 ± 3.3	46.5 ± 10.2^*^	23.9 ± 4.2^*#^
Trp (μg/ml) (mean ± SD)	4.97 ± 1.55	5.25 ± 1.52	5.90 ± 1.60^*#^
KYN (μg/ml) (mean ± SD)	0.34 ± 0.12	0.37 ± 0.15	0.40 ± 0.11^*^
KYNA (ng/ml) (mean ± SD)	3.28 ± 1.84	3.79 ± 2.03^*^	3.88 ± 1.39^*^
ANA (ng/ml) (mean ± SD)	2.28 ± 2.14	3.76 ± 3.09^*^	3.29 ± 2.88
3-HANA (ng/ml) (mean ± SD)	9.11 ± 4.33	4.70 ± 3.86^*^	6.67 ± 2.89^*#^
QUINA (ng/ml) (mean ± SD)	18.2 ± 12.85	10.8 ± 9.25^*^	15.3 ± 10.2^#^
5-HIAA (ng/ml) (mean ± SD)	32.6 ± 11.4	34.5 ± 13.3	37.0 ± 10.5
XA (ng/ml) (mean ± SD)	1.83 ± 1.0	0.89 ± 0.56^*^	1.10 ± 0.66^*^
3-HK (ng/ml) (mean ± SD)	1.81 ± 1.70	0.74 ± 1.26^*^	2.70 ± 2.50^#^

p < 0.05 vs. healthy controls (*) or vs. patients affected by schizophrenia (#) (Mann-Whitney/Chi-squared test). (a) DSM-V codes.

BMI–Body Mass Index; CGI–Clinical Global Impression; GAF–Global Assessment of Functioning; PANSS–Positive and Negative Syndrome Scale for Schizophrenia. For abbreviation of kynurenine-metabolites, see main text.

**Table 2:** Serum levels of xanthurenic acid (XA) and other kynurenine metabolites in the subgroups of FES and MES patients, as compared to healthy controls.

	Controls (n = 84)	FES (n = 30)	MES (n = 60)
Gender (F; n,%)	47 (55.9)	10 (33.3)^*^	17 (28.3)^*^
Age (years; mean ± SD)	32.8 ± 10.4	26.9 ± ± 9.0^*#^	36.7 ± 11.6^*^
BMI (mean ± SD)	23.1 ± 4.0	23.8 ± 3.5^*^	25.0 ± 4.3^*^
Antipsychotic treatment (n,%)	–	0 (0.0)	60 (100.0)^*^
Substance Abuse (n,%)	6 (7.1)	11 (36.7)^*^	16 (26.7)^*^
Alcohol Abuse (n,%)	6 (7.1)	13 (43.3) ^*#^	6 (10.0)
Cigarettes smoking (n,%)	27 (32.1)	23 (76.7)^*^	40 (66.7)^*^
CGI (mean ± SD)	1.0 ± 0.0	5.1 ± 1.0^*^	5.4 ± 1.0^*^
GAF (mean ± SD)	93. 0 ± 2.8	39.7 ± 16.1^*^	41.3 ± 13.4^*^
PANSS tot (mean ± SD)	32.6 ± 3.3	95.0 ± 12.8^*^	89.5 ± 19.8^*^
PANSS positive (mean ± SD)	7.0 ± 0.0	22.8 ± 6.7^*#^	18.2 ± 7.1^*^
PANSS negative (mean ± SD)	7.0 ± 0.0	21.8 ± 5.8^*#^	27.2 ± 7.4^*^
PANSS general (mean ± SD)	18.6 ± 3.3	50.4 ± 8.6^*#^	44.6 ± 10.4^*^
Trp (μg/ml) (mean ± SD)	4.97 ± 1.55	5.01 ± 1.38	5.37 ± 1.58
KYN (μg/ml) (mean ± SD)	0.34 ± 0.12	0.39 ± 0.18	0.36 ± 0.12
KYNA (ng/ml) (mean ± SD)	3.28 ± 1.84	3.99 ± 2.22	3.69 ± 1.93
ANA (ng/ml) (mean ± SD)	2.28 ± 2.14	4.81 ± 3.48^*#^	3.24 ± 2.75^*^
3-HANA (ng/ml) (mean ± SD)	9.11 ± 4.33	2.63 ± 1.93^*#^	5.77 ± 4.17^*^
QUINA (ng/ml) (mean ± SD)	18.2 ± 12.85	10.7 ± 8.65^*^	10.9 ± 9.63^*^
5-HIAA (ng/ml) (mean ± SD)	32.6 ± 11.4	35.7 ± 16.1	34.0 ± 11.8
XA (ng/ml) (mean ± SD)	1.83 ± 1.0	0.76 ± 0.37^*^	0.96 ± 0.62^*^
**3-HK (ng/ml) (mean** ± **SD)**	1.81 ± 1.70	0.31 ± 0.17^*#^	0.96 ± 1.50^*^

p < 0.05 vs. healthy controls (*) or vs. MES patients (#) (Mann-Whitney/Chi- squared test). test). (a) DSM-V codes.

**FES –** first episode schizophrenia; **MES –** multiple episode schizophrenia; **BMI**– Body Mass Index; **CGI** – Clinical Global Impression; **GAF** – Global Assessment of Functioning; **PANSS** – Positive and Negative Syndrome Scale for Schizophrenia. For abbreviation of kynurenine metabolites, see main text.

**Table 3:** Serum levels of xanthurenic acid (XA) and other kynurenine metabolites in FES patients before T_0_ and after 12 months of treatment (T_1_) with antipsychotic drugs.

	FES T_0_ (n = 14)	FES T_1_ (n = 14)
CGI (mean ± SD)	5.0 ± 1.10	3.4 ± 0.9^*^
GAF (mean ± SD)	42.1 ± 18.2	60.1 ± 15.5^*^
PANSS tot (mean ± SD)	93.9 ± 12.9	70.6 ± 24.7^*^
PANSS positive (mean ± SD)	23.1 ± 5.8	14.8 ± 7.2^*^
PANSS negative (mean ± SD)	21.8 ± 3.7	18.7 ± 6.8
PANSS general (mean ± SD)	49.0 ± 8.8	37.1 ± 12.9^*^
New Antipsychotic treatment		
Risperidone (n,%)	5 (35.7)	–
Paliperidone (n,%)	2 (14.3)	–
Olanzapine (n,%)	1 (7.1)	–
Aripiprazole (n,%)	4 (28.6)	–
Clozapine (n,%)	2 (14.3)	–
Trp (μg/ml) (mean ± SD)	5.06 ± 1.28	3.88 ± 1.13^*^
KYN (μg/ml) (mean ± SD)	0.35 ± 0.21	0.41 ± 0.10^*^
KYNA (ng/ml) (mean ± SD)	3.96 ± 2.98	3.9 ± 1.68
ANA (ng/ml) (mean ± SD)	5.03 ± 3.32	3.38 ± 3.41
3-HANA (ng/ml) (mean ± SD)	2.20 ± 1.78	7.95 ± 5.78^*^
QUINA (ng/ml) (mean ± SD)	9.18 ± 6.07	10.33 ± 5.59
5-HIAA (ng/ml) (mean ± SD)	32.30 ± 17.30	37.10 ± 8.44^*^
XA (ng/ml) (mean ± SD)	0.75 ± 0.40	0.95 ± 0.53
3-HK (ng/ml) (mean ± SD)	0.29 ± 0.09	1.64 ± 2.07^*^

Wilcoxon signed rank test (H0: no differences between baseline and 1-year follow-up values normalized by the baseline values). *p < 0.05 vs. values at T_1_. CGI – Clinical Global Impression; GAF – Global Assessment of Functioning; PANSS – Positive and Negative Syndrome Scale for Schizophrenia. For abbreviation of kynurenine metabolites, see main text.
