# Supplementary material for: Xanthurenic Acid Activates mGlu2/3 Metabotropic Glutamate Receptors and is a Potential Trait Marker for Schizophrenia
Source: Sci Rep. 2015 Dec 8;5:17799. doi: 10.1038/srep17799 (PMC4672300; doi:10.1038/srep17799)
Supplement: Supplementary Information [file srep17799-s1.doc]

# XANTHURENIC ACID ACTIVATES mGlu2/3 METABOTROPIC GLUTAMATE RECEPTORS AND IS A POTENTIAL TRAIT MARKER FOR SCHIZOPHRENIA

**Francesco Fazio, Luana Lionetto, Martina Curto, Luisa Iacovelli, Michele Cavallari, Cristina Zappulla, Martina Ulivieri, Flavia Napoletano, Matilde Capi, Valentina Corigliano, Sergio Scaccianoce, Alessandra Caruso, Jessica Miele, Antonio De Fusco, Luisa Di Menna, Anna Comparelli, Antonella De Carolis, Roberto Gradini, Robert Nisticò, Antonio De Blasi, Paolo Girardi, Valeria Bruno, Giuseppe Battaglia, Ferdinando Nicoletti, and Maurizio Simmaco.**

**Supplementary Information**

## Methods

*Materials*

Xanthurenic acid (XA), cytosine β-D-arabinofuranoside (AraC), glutamate and glycine and 3- isobutyl-1-methylxanthine (IBMX) were purchased from Sigma-Aldrich (St. Louis, MO). Forskolin, L-(+)-2-amino-4-phosphonobutyric acid (L-AP4), N-methyl-D-aspartate (NMDA), kynurenic acid (KYNA), 3,5-dihydroxyphanylglycine (DHPG), 2R,4R-2-aminopyrrolidin-2,4- dicarboxylate (2R,4R-APDC), 1S,2R,5R,6R)-2-amino-4-oxabicyclo[3.1.0]hexane-2,6-dicarboxylic acid (LY379268), 2-[(1*S*,2*S*)-2-carboxycyclopropyl]-3-(9*H*-xanthen-9-yl)-D-alanine (LY341495) and were purchased from Tocris Cookson (Anawa Trading SA, Zurich, Switzerland; and Bristol, U.K.). Tissue culture reagents were from Invitrogen (Milan, Italy). Xanthurenic acid [ring-5,7-3H]

(40-60 Ci/mmol) and (2S)-2-amino-2-[(1S,2S)-2-carboxycycloprop-1-yl]-3-[2,7-3H]xanth-9-yl)

propanoic acid ([^3^H]LY341495) were purchased from American Radiolabeled Chemicals, Inc. (St. Louis, MO). N-4′-cyano-biphenyl-3-yl)-N-(3-pyridinylmethyl)-ethanesulfonamide hydrochloride (LY566332) was kindly provided by Eli Lilly and Company (Indianapolis, IN).

*Cell Transfection*

In brief, human embryonic kidney (HEK293) cells were transfected using 8 µl of LipofectAMINE2000in OptiMEM medium (Invitrogen, Carlsbad, CA), and 10 µg of cDNA for 4 h. mGlu2 receptor cDNA was kindly provided by J. Blahos (Academy of Science, Prague, Czech Republic); mGlu3 receptor cDNA was kindly provided by F. Ferraguti (Innsbruck Medical University, Innsbruck, Austria); mGlu4 receptor cDNA was kindly provided by J.P. Pin (Institute

du Génomique Fonctionelle, Montpellier, France). Cells were co-transfected with 2.5 µg/dish of type-V adenylyl cyclasecDNA^1^.

*Measurement of cAMP formation in HEK293 cells*

Cultures were incubated in Hanks' balanced salt solution buffer, pH 7.4, containing 0.5 mM IBMX and bovine serum albumin (0.3%). 2R, 4R-APDC (100 μM), LY341495 (1 μM) or XA were added 3 min prior to forskolin (1 μM). After 20 min, the reaction was terminated with ice-cold ethanol. cAMP levels were measure by RIA (PerkinElmer Life and Analytical Sciences, Waltham, MA).

*Measurement of cAMP formation in cortical slices*

We examined the effect of XA and LY37928 in cortical slices prepared from adult mGlu2-/- mice and their age-matched wt counterparts following the procedure described by Iacovelli et al., 20092, with the difference that here cAMP formation was measured using an ELISA kit (Tema Ricerche,

Italy). In brief, 350 x 350 μm slices were prepared from the cerebral cortex and incubated in Krebs- Hensleit buffer at 37°C under constant oxygenation to allow metabolic recovery. Forty μl of gravity-packed slices were then incubated in the presence of 0.5 mM IBMX for 15 min. After the

addition of forskolin and/or XA or LY379268 (applied 2 min prior to forskolin), and LY341495 (the latter applied 1 min prior to XA or LY379268) the incubation was continued for 20 min. The reaction was stopped with 0.4 N HClO_4_.

*Measurement of polyphosphoinositide (PI) hydrolysis in cortical slices*

Receptor agonist-stimulated PI hydrolysis was measured in adult mouse cortical slices. Slices (see above) were incubated at 37°C under constant oxygenation for 30 to 45 min in Krebs-Hensleit buffer equilibrated with 95% O2 / 5% CO2 to pH 7.4. Forty μl of gravity-packed slices were then incubated for 60 min in 250 μl of buffer containing 1 μCi of [myo-^3^H]inositol. Slices were incubated with LiCl (10 mM for 10 min) followed by the indicated concentrations of DHPG and/or XA or LY379268. One hour later, the incubation was stopped by the addition of 900 μl of

methanol/chloroform (2:1), after washing the slices with ice-cold buffer. After further addition of 300 μl of chloroform and 600 μl of water, samples were centrifuged at low speed to facilitate phase separation, and the upper aqueous phase was loaded into Dowex 1-X-8 columns (Dow Chemical Company, Midland, MI). For the separation of [^3^H]InsP, columns were washed twice with water, once with a solution of 5 mM sodium tetraborate and 40 mM sodium formate to elute cyclic InsP and glycerophosphoinositols, and then with 6.5 ml of 0.2 M ammonium formate and 0.1 M formic acid for the elution of InsP.

*Binding studies*

1. Mouse cerebral cortex

[^3^H]LY341495 binding was assessed using the conditions described by Johnson et al., 19993, with the difference that KCl was used instead of KBr in the assay buffer (see below). The same conditions were used for measurements of [^3^H]XA binding. Mouse cerebral cortex was homogenized (10% w/v) in: 0.32 M sucrose, 1mM NaHCO3, 1 mM MgCl2, 0.5mM CaCl2 (buffer

A) with a Teflon-glass homogenizer. The homogenate was centrifuged for 10 min at 1500 x g, and the resulting pellet (P1) was washed once with buffer A by homogenization and centrifugation for

10 min at 755 x g. The combined supernatants were centrifuged for 10 min at 17,300 x g and the resulting crude membrane preparation (P2 fraction) was frozen at -80 °C. Membranes were washed three times with ice-cold assay buffer (10 mM potassium phosphate and 100 mM potassium chloride, pH 7.6); an aliquot of tissue was used for protein determinations. Proteins (100 μg) were incubated in ice-cold assay buffer for 60 min in the presence of [^3^H]XA (5 nM) or [^3^H]LY341495 (5nM) and the indicated concentrations of non-radioactive compounds. Membranes were separated by rapid filtration with 5 x 1 ml of ice-cold assay buffer using Whatman GF-B filters (Sigma- Aldrich).

b) HEK293 cells

Binding experiments were performed 72 h after transfection. Cells were scraped and homogenized in buffer A (0.32 M sucrose, 1mM NaHCO3, 1 mM MgCl2, 0.5mM CaCl) using a Teflon-glass homogenizer. The homogenate was centrifuged for 10 min at 1500 x g, at 4°C. The supernatant was kept on ice and the resulting pellet (P1) was washed once and homogenized in buffer A and then centrifuged for 10 min at 755 x g, at 4°C. The two supernatants were combined and centrifuged for 10 min at 17,000 x g and the resulting crude membrane preparation (P2 fraction) was washed three times with ice-cold assay buffer (10 mM potassium phosphate and 100 mM potassium chloride, pH 7.6). After protein determination, membranes (100-200 μg/sample) were incubated in ice-cold assay buffer for 60 min in the presence of [^3^H]-XA (5 nM) and the indicated concentrations of non-radioactive compounds. Incubations were stopped by adding 5 ml of ice-cold assay buffer and rapid filtration through GF/C glass fibre filters (Whatman) with two sequential 5 ml washes.

*MK-801-induced increase in locomotor activity*

Locomotor activity was monitored using boxes (42 x 42 x 21 cm) in conjunction with Activity Monitor equipped with infrared photobeam interruption sensor and a computerised analysis system (Open Field Activity System Hardware; Med Associates, Inc. PO Box 319, St. Albans).

Mice (mGlu2-/- and their wt counterparts) were pre-treated with saline or XA (30 or 60 mg/kg),

individually placed into the boxes and allowed to habituate to the cage for 60 minutes. Following the habituation phase, mice received MK801 (0.32 mg/Kg) or saline and maintained in the cage for additional 180 min. The first 5 min following MK-801 injection were not included in the statistical analysis.

*LC/MS-MS methods for the assay of kynurenine metabolites Chemical and reagents*

Standards of Trp, 5-HIAA, KYN, KYNA, 3-HANA, ANA, XA, 3-HK, QUINA, and internal standard (IS) dansyl-norvaline (DNSnVal) were purchased from Sigma Aldrich. HPLC-grade methanol was purchased from Carlo Erba reagents (Milan, Italy) and formic acid was from Merck (Darmstadt, Germany). Water was deionized and filtered with a Milli-Q Plus apparatus (Millipore Corporation, Bedford, MA, USA).

*Stock solutions and working standard*

Stock solutions (1mg/mL) of analytes were prepared by dissolving the pure analytes in solubilization solvent for each analyte. Working solutions were prepared by diluting stock solutions with deionised water in order to obtain a final concentration of 100 µg/mL for Trp and KYN, and

- 1. µg/ml for KYNA, ANA, 3-HK, XA, 3-HANA, QUIN and 5-HIAA. To prepare standard curve, working solutions were diluted to generate calibration levels covering a range of 0.5-20 µg/ml for Trp and KYN, 0.2-20.0 ng/ml for KYNA, ANA, 3-HK and XA, and 1.0-50.0 ng/ml for 3-HANA, QUIN and 5-HIAA. QC samples were prepared following the same procedure giving low, medium and high analyte concentrations. Working solution of DNSnVal (50 µM) was prepared in acetonitrile for serum deproteinisation. All solutions were stored at −20 °C until use. For each analyte, serial dilutions at the concentrations of linearity range were obtained (Table S1). Calibration standard samples, QC samples and serum samples were processed in the same way.

*Samples storage and preparation*

For all patients we obtained blood samples for determining the kynurenine pathway metabolite serum levels. After centrifugation at 1500 *g* for 10 min, aliquots of serum were stored at -80°C until analysis. 100 μl of serum samples were added to 100 μl of IS working solution (100µM in TCA 4%). The samples were vortex-mixed for 10 sec and centrifuged at 14000 *g* for 5 min. One hundred μl of clean upper layer were transferred to a vial for the autosampler. Eighty μl were injected into the chromatographic system.

*Chromatographic conditions*

The HPLC analysis was performed using an Agilent Liquid Chromatography System series 1100 (Agilent Technologies, USA) which included a binary pump, an autosampler, a solvent degasser, and a column oven. Chromatographic separation was performed on a pentafluorophenyl column (100x2.1 mm, Kinetex PFP, 2.6 μm, 100 Å pore size, Phenomenex, CA, USA) equipped with a security guard precolumn (Phenomenex, Torrance, CA) containing the same packing material. The column was maintained at room temperature. The mobile phase consisted of a solution of 0.1% aqueous formic acid (eluent A) and 100% of methanol (eluent B); elution was performed at flow rate of 300 μl/min, using an elution gradient. The injection volume was 80 μl, and the total analysis run time was 16 min.

*Mass Spectrometry Conditions*

The mass spectrometry method was performed on a 3200 triple quadrupole system (Applied Biosystems, Foster City, CA) equipped with a Turbo Ion Spray source. The detector was set in the positive ion mode. The ion spray voltage was set at 5000 V and the source temperature was 300 °C, the collision activation dissociation (CAD) gas was set at medium value, and nitrogen was used as collision gas. The Q1 and Q3 quadrupoles were tuned for the unit mass resolution. The transition of the precursor ions to product ions was monitored with a dwell time of 100 ms for each analyte. The instrument was set in the multiple reaction monitoring (MRM) mode. Mass spectrometer parameters were optimized to maximize sensitivity for all transition (Table S3). Calibration curves were obtained using Analyst from data generated with the calibration samples plotting the analyte to

IS peak area ratios (PARs) against concentration by a weighted (1/x^2^) least-squares linear

regression. The PARs of unknown samples were interpolated on the calibration curve to obtain the analytes concentrations. Data were acquired and processed by Analyst 1.5.1 Software.

*Assay validation*

The method performance was assessed through the qualifier and the quantifier transitions. Specific MS/MS conditions for each analyte were optimized using a 1µg/mL solution for each analyte. A flow rate of 10 µl/min was used. A full validation included limit of detection (LOD), lower limit of quantification (LLOQ), linearity, recovery, within-day and between-day precision and accuracy.

LOD was defined as the lowest detectable amount of analytes that gives a signal-to-noise ratio of 5. The LLOQ was defined as that amount of analytes that can still be determined with acceptable precision (relative SD < 20%, RSD) and accuracy (%deviation < 20%, RE). The linearity of calibration curves was established analyzing standard mixtures at different values. Recoveries were evaluated by analyzing five different samples in triplicate before and after enrichment with three different concentrations of each analyte. Recovery was calculated as follows: (final concentration – initial concentration)/added concentration. Accuracy and precision of the method were assessed from the results of standard mixtures analyzed thrice in quintuplicate. The within-day precision (%RSD) and accuracy (%RE) were determined by measuring seven concentrations of analytes in five batches of triplicates within a day. The between-day precision and accuracy were calculated by analyzing seven concentrations of analytes in triplicates for five consecutive days.

*LC–MS/MS parameters*

The MS/MS conditions, which consist of ion fragments, declustering potential, entrance potential, collision energy and collision cell exit potential for all analytes are reported in Table S3. Method optimization used the most intense precursor/product transitions for each metabolite. The collision energy voltage, fragmentation voltage and capillary voltage were adjusted to provide the highest sensitivity. All the analytes were analyzed with MRM scan. This LC-MS/MS method enables the measurement of kynurenine metabolites with the following retention times: Trp (4.92 min), KYN (1.96 min), KYNA (8.16 min), 3-HK (1.41 min), ANA (6.67 min), 3-HANA (2.87 min), XA (8.42 min), QUINA (1.20 min), 5-HIAA (7.50 min), IS (9.58 min).

*Linearity and detection limits*

The developed method was validated considering the limit of detection (LOD), lower limit of quantification (LLOQ), linear dynamic range, recovery, precision and accuracy. Since all analytes are endogenous metabolites, the determination of LOD and LLOQ of the method was carried out

using standard mixtures. LOD for each analyte was calculated by considering a signal-to-noise ratio of 5. LLOQ was estimated by considering a signal to-noise ratio of 15. The results indicated that the LOD was in the range of 0.05 ng/ml and the LLOQ level was between 0.25 and 1.25 ng/ml, showing that the developed method is highly sensitive for the simultaneous quantification of the targeted analytes. Eight-point calibration curve was found linear over the concentration range of 0.1-20.0 µg/ml for Trp, 0.05-5µg/ml for KYN, 0.5-200.0 ng/ml for KYNA, 3-HK, ANA and XA, and 1.0-200.0 ng/ml for 3-HANA, QUINA and 5-HIAA. Calibration graphs were constructed based on the peak area ratio (analyte/IS) versus the spiked concentrations by least square linear regression analysis with a weighting factor of 1/x2. The mean correlation coefficient of the weighted calibration curve generated during the validation was >0.96 (Table S1).

*Precision and accuracy*

The accuracy and precision of each point on the standard curve were <15% RE and RSD and were used to calculate the accuracy and precision of the method. Standard mixtures were analyzed 3 times in quintuplicate. The within-day precision and accuracy were obtained by measuring seven concentrations of analytes in five batches of triplicates within a day. The between-day precision and accuracy were evaluated on five consecutive days in triplicates. The results are shown in Table S3. Recoveries were evaluated by analyzing five different samples in triplicate before and after enrichment with three different concentrations of each analyte (low, medium, high). Mean recovery of each analyte was calculated by averaging the recoveries obtained at the three assayed concentrations and resulted as follows (mean ± SD; %): Trp: 98.21±1.86; KYN: 95.39±3.90; KYNA: 93.76±6.43; 3-HK: 95.31±4.29; ANA: 94.31±1.52; 3-HANA: 97.08±3.43; XA: 94.98±3.36;

QUINA: 97.84±2.67; 5-HIAA 98.55±2.05 (Table S3).

***Supplementary Results***

*Correlations between serum levels of kynurenine metabolites and clinical scales in patients affected by schizophrenia*

In MES patients, we found weak but significant correlations. KYN and KYNA levels were negatively correlated with PANSS positive scale scores and KYN and 5-HIAA levels were positively correlated with GAF scores. QUINA and Trp levels were negatively correlated with CGI and PANSS negative scores, respectively (Table S5). We extended the correlation analysis to the seven cognitive domains of the Neuropsychological Test Battery (see Table S6) in a subgroup of patients including 25 FES and 18 MES patients. In the overall population of patients, we found only a negative correlation between KYN or 5-HIAA levels and speed of processing, and a negative correlation between 3-HK levels and reasoning. Trp levels positively correlated with working memory (Table S7). In FES patients, unexpectedly, KYN, KYNA, QUINA and 5-HIAA levels were all negatively correlated with speed or processing, whereas 3-HANA levels were negatively correlated with sustained attention and working memory. In contrast, Trp levels were positively correlated with scores of most cognitive domains (Table S8). In MES patients, 3-HK and ANA levels were negatively correlated with reasoning and visual learning, respectively. 5-HIAA levels were positively correlated with reasoning (Table S9). XA levels did not correlate with any clinical and cognitive measurements in patients affected by schizophrenia.

# Supplementary References

- - 1. Aramori, I. *et al*. Molecular mechanism of desensitization of the chemokine receptor CCR- 5: receptor signaling and internalization are dissociable from its role as an HIV-1 co- receptor. *EMBO J.* **16**, 4606-4616 (1997).
    2. Iacovelli, L. *et al*. Regulation of group II metabotropic glutamate receptors by G protein- coupled receptor kinases: mGlu2 receptors are resistant to homologous desensitization. *Mol. Pharmacol*. **75**, 991-1003 (2009).
    3. Johnson, B. G. *et al*. [^3^H]-LY341495 as a novel antagonist radioligand for group II metabotropic glutamate (mGlu) receptors: characterization of binding to membranes of mGlu receptor subtype expressing cells. *Neuropharmacology.* **38**, 1519-1529 (1999).

**Table S1**. Sensitivity and linearity parameters of the LC-MS/MS method for the assay of kynurenine metabolites.

| **Compound** | **LOD (ng/ml)** | **LOQ (ng/ml)** | **Linearity Range** | ***R*** |
| --- | --- | --- | --- | --- |
| **Trp** | 0.5 | 1.25 | 0.1-20μg/ml | 0.9964 |
| **KYN** | 0.1 | 0.25 | 0.05-5μg/ml | 0.9930 |
| **KYNA** | 0.1 | 0.25 | 0.05-50 ng/ml | 0.9958 |
| **3-HK** | 0.1 | 0.25 | 0.05-50ng/ml | 0.9609 |
| **ANA** | 0.1 | 0.25 | 0.05-50ng/ml | 0.9955 |
| **3-HANA** | 0.5 | 1.25 | 0.05-50ng/ml | 0.9855 |
| **XA** | 0.15 | 0.6 | 0.05-50 ng/ml | 0.9932 |
| **QUINA** | 0.25 | 1.0 | 0.05-50ng/ml | 0.9904 |
| **5-HIAA** | 0.5 | 1.0 | 0.25-100ng/ml | 0.9808 |

For abbreviations of kynurenine metabolites, see main text.

**Table S2**. Mass spectrometry parameters.

| **Analyte** | **Precursor Ion (m/z)** | **Fragments (m/z)** | **DP (V)** | **EP (V)** | **CE (V)** | **CXP (V)** |
| --- | --- | --- | --- | --- | --- | --- |
| **IS** | 351,15 | 170.0 | 42 | 7.0 | 26 | 2.6 |
| **Trp** | 204.7 | 159.0 | 5.0 | 4.6 | 21.0 | 2.5 |
|  |  | 146.2 | 5.0 | 4.6 | 27.0 | 2.3 |
|  |  | 118.1 | 5.0 | 4.6 | 33.0 | 2.3 |
| **KYN** | 209.0 | 174.0 | 33.2 | 4.2 | 22.0 | 2.5 |
|  |  | 146.1 | 33.2 | 4.2 | 22.1 | 2.5 |
|  |  | 136.1 | 33.2 | 4.2 | 16.7 | 2.4 |
| **KYNA** | 190.0 | 144.1 | 37.9 | 4.9 | 23.3 | 2.4 |
|  |  | 162.1 | 37.9 | 4.9 | 22.9 | 2.8 |
|  |  | 116.0 | 37.9 | 4.9 | 41.7 | 2.3 |
| **3-HK** | 225.1 | 190.0 | 26.0 | 5.2 | 19.5 | 2.9 |
|  |  | 162.1 | 26.0 | 5.2 | 25.0 | 2.8 |
|  |  | 152.1 | 26.0 | 5.2 | 17.1 | 2.5 |
|  |  | 110.2 | 26.0 | 5.2 | 24.2 | 2.4 |
| **ANA** | 138.2 | 120.1 | 28.9 | 4.5 | 15.7 | 2.3 |
|  |  | 92.1 | 28.9 | 4.5 | 27.9 | 2.9 |
| **3-HANA** | 154.1 | 108.2 | 34.0 | 5.2 | 29.0 | 2.9 |
|  |  | 80.1 | 34.0 | 5.2 | 36.8 | 2.8 |
| **XA** | 206.0 | 178.2 | 35.4 | 7.7 | 23.1 | 4.0 |
|  |  | 160.1 | 35.4 | 7.7 | 26.6 | 2.5 |
|  |  | 132.2 | 35.4 | 7.7 | 38.8 | 2.3 |
| **QUINA** | 168.1 | 124.1 | 20.0 | 5.7 | 16.7 | 3.8 |
|  |  | 106.2 | 20.0 | 5.7 | 20.0 | 3.3 |
|  |  | 78.1 | 20.0 | 5.7 | 33.0 | 2.5 |
| **5-HIAA** | 192.0 | 146.1 | 31.0 | 5.0 | 18.0 | 2.4 |
|  |  | 118.2 | 31.0 | 5.0 | 38.0 | 3.7 |

**IS**–Internal Standard; **DP**–Declustering Potential; **EP**–Entrance Potential; **CE**– Collision Energy; **CXP**–Collision Cell Exit Potential. For abbreviation of

kynurenine metabolites, see main text.

**Table S3**. Within- and between-day precision and accuracy of the LC-MS/MS method

| **Analyte** | **Concentration added** | *Within-day* | | | *Between-day* | | |
| --- | --- | --- | --- | --- | --- | --- | --- |
|  |  | **Concentration found (mean±SD)** | **Precision (RSD%)** | **Accuracy (RE%)** | **Concentration found (mean±SD)** | **Precision (RSD%)** | **Accuracy (RE%)** |
| **Trp (μg/mL)** | 0.1 | 0.09±0.00 | 4.4 | 5.4 | 0.10±0.00 | 1.3 | 4.5 |
|  | 0.625 | 0.62±0.041 | 1.9 | 0.9 | 0.62±0.01 | 1.9 | 0.5 |
|  | 1.25 | 1.24±0.05 | 4.2 | 1.1 | 1.23±0.08 | 6.8 | 1.8 |
|  | 2.5 | 2.43±0.12 | 4.7 | 2.7 | 2.46±0.15 | 5.9 | 1.5 |
|  | 5 | 4.98±0.21 | 4.2 | 0.3 | 4.98±0.21 | 4.3 | 0.3 |
|  | 10 | 9.94±0.27 | 2.7 | 0.6 | 9.96±0.26 | 2.6 | 0.4 |
|  | 20 | 19.80±0.12 | 0.6 | 1.0 | 19.74±0.17 | 0.9 | 1.3 |
| **KYN**  **(ng /mL)** | 10 | 9.90±0.20 | 1.5 | 0.5 | 9.90±0.20 | 2.4 | 0.8 |
|  | 25 | 24.7±0.4 | 1.7 | 1.2 | 24.6±0.5 | 2.0 | 1.8 |
|  | 50 | 49.7±0.6 | 1.3 | 0.7 | 49.9±0.4 | 1.2 | 0.3 |
|  | 75 | 74.6±1.8 | 2.5 | 0.5 | 74.1±1.5 | 2.0 | 1.3 |
|  | 100 | 103.0±6.4 | 6.2 | -3.0 | 100.3±1.9 | 1.9 | -0.3 |
|  | 500 | 501.4±16.4 | 3.3 | -0.3 | 501.9±5.5 | 1.1 | -0.4 |
|  | 1000 | 999.0±22.0 | 2.2 | 0.1 | 999.3±20.2 | 2.0 | 0.1 |
| **KYNA**  **(ng/mL)** | 1 | 0.98±0.03 | 2.6 | 2.5 | 0.99 ±0.02 | 2.3 | 1.3 |
|  | 2.5 | 2.49±0.03 | 1.2 | 0.4 | 2.47±0.03 | 1.1 | 1.1 |
|  | 5 | 4.93±0.06 | 1.3 | 1.3 | 4.95±0.07 | 1.4 | 1.0 |
|  | 7.5 | 7.44±0.08 | 1.1 | 0.8 | 7.44±0.07 | 1.0 | 0.9 |
|  | 10 | 9.84±0.13 | 1.3 | 1.6 | 9.87±0.14 | 1.5 | 1.3 |
|  | 50 | 49.55±0.66 | 1.3 | 0.9 | 49.64±0.64 | 1.3 | 0.7 |
|  | 100 | 98.62±0.83 | 0.8 | 1.4 | 99.05±0.93 | 0.9 | 1.0 |
| **3-HK**  **(ng/mL)** | 1 | 0.98±0.03 | 2.8 | 1.6 | 1.00±0.04 | 4.1 | -0.1 |
|  | 2.5 | 2.45±0.05 | 2.2 | 2.0 | 2.46±0.05 | 2.2 | 1.5 |
|  | 5 | 4.90±0.09 | 1.8 | 2.0 | 4.90±0.10 | 2.0 | 2.1 |
|  | 7.5 | 7.42±0.07 | 0.9 | 1.1 | 7.44±0.13 | 1.7 | 0.8 |
|  | 10 | 9.88±0.11 | 1.1 | 1.2 | 9.85±0.16 | 1.6 | 1.5 |
|  | 50 | 49.75±0.32 | 0.7 | 0.5 | 49.66±0.77 | 1.6 | 0.7 |
|  | 100 | 99.67±0.79 | 0.8 | 0.3 | 99.61±0.74 | 0.7 | 0.4 |
| **ANA**  **(ng/mL)** | 1 | 0.97±0.04 | 3.9 | 5.9 | 0.97±0.03 | 3.4 | 2.6 |
|  | 2.5 | 2.43±0.06 | 2.7 | 2.8 | 2.47±0.06 | 2.4 | 1.2 |
|  | 5 | 4.88±0.12 | 2.5 | 2.4 | 4.94±0.18 | 3.8 | 1.3 |
|  | 7.5 | 7.33±0.09 | 1.2 | 2.2 | 7.42±0.11 | 1.5 | 1.1 |
|  | 10 | 9.90±0.07 | 0.7 | 1.0 | 9.83±0.14 | 1.5 | 1.7 |
|  | 50 | 49.95±0.08 | 0.2 | 0.1 | 49.94±0.55 | 1.1 | 0.1 |
|  | 100 | 99.19±1.35 | 1.4 | 0.8 | 99.58±1.09 | 1.1 | 0.4 |
| **3-HANA**  **(ng/mL)** | 1 | 0.98±0.03 | 3.4 | 2.1 | 1.00±0.02 | 2.4 | -0.3 |
|  | 2.5 | 2.47±0.05 | 2.1 | 1.3 | 2.43±0.07 | 2.7 | 2.9 |
|  | 5 | 4.95±0.11 | 2.3 | 1.0 | 4.93±0.12 | 2.4 | 1.4 |
|  | 7.5 | 7.37±0.11 | 1.5 | 1.8 | 7.49±0.13 | 1.7 | 0.1 |
|  | 10 | 9.79±0.15 | 1.5 | 2.1 | 9.86±0.13 | 1.3 | 1.4 |
|  | 50 | 49.21±0.51 | 1.1 | 1.6 | 49.48±0.65 | 1.3 | 1.1 |
|  | 100 | 98.70±1.19 | 1.2 | 1.3 | 98.85±1.94 | 2.0 | 0.0 |
| **XA** | 1 | 0.99±0.01 | 1.0 | 1.2 | 0.99±0.01 | 1.0 | 1.1 |
| **(ng/mL)** | 2.5 | 2.50±0.05 | 2.0 | 0.1 | 2.49±0.06 | 2.4 | 0.5 |
|  | 5 | 4.94±0.07 | 1.4 | 1.3 | 4.97±0.07 | 1.5 | 0.6 |
|  | 7.5 | 7.48±0.08 | 1.1 | 0.3 | 7.49±0.07 | 1.0 | 0.1 |
|  | 10 | 9.94±0.06 | 0.6 | 0.6 | 9.93±0.06 | 0.6 | 0.7 |
|  | 50 | 49.92±0.09 | 0.2 | 0.2 | 50.03±0.09 | 0.2 | -0.1 |
|  | 100 | 99.95±0.09 | 0.1 | 0.1 | 100.02±0.12 | 0.1 | -0.1 |
| **QUINA**  **(ng/mL)** | 1 | 0.96±0.06 | 5.8 | 4.0 | 0.99±0.05 | 4.6 | 1.1 |
|  | 2.5 | 2.46±0.07 | 2.7 | 1.6 | 2.47±0.11 | 4.5 | 1.1 |
|  | 5 | 4.97±0.15 | 2.9 | 0.6 | 4.85±0.15 | 3.0 | 2.9 |
|  | 7.5 | 7.46±0.09 | 1.2 | 0.5 | 7.43±0.09 | 1.2 | 1.0 |
|  | 10 | 9.81±0.17 | 1.7 | 1.9 | 10.06±0.24 | 2.4 | -0.6 |
|  | 50 | 49.06±0.85 | 1.7 | 1.9 | 49.06±0.94 | 1.9 | 1.9 |
|  | 100 | 99.55±0.64 | 0.6 | 0.5 | 99.26±0.39 | 0.4 | 0.7 |
| **5-HIAA**  **(ng/mL)** | 1 | 0.98±0.01 | 0.7 | 1.6 | 0.98±0.04 | 3.6 | 2.0 |
|  | 2.5 | 2.46±0.06 | 2.3 | 1.7 | 2.47±0.05 | 2.2 | 1.3 |
|  | 5 | 4.91±0.09 | 1.9 | 1.9 | 4.93±0.11 | 2.2 | 1.4 |
|  | 7.5 | 7.45±0.13 | 1.7 | 0.6 | 7.49±0.10 | 1.3 | 0.1 |
|  | 10 | 9.87±0.16 | 1.6 | 1.3 | 9.99±0.15 | 1.6 | 0.2 |
|  | 50 | 48.99±0.87 | 1.8 | 2.0 | 49.59±1.06 | 2.1 | 0.8 |
|  | 100 | 99.00±2.84 | 2.9 | 1.0 | 100.03±1.92 | 1.9 | 0.1 |
| **Mean recovery (mean ± SD; %).** Trp: 98.21±1.86; Kyn: 95.39±3.90; KYNA: 93.76±6.43; 3-HK: 95.31±4.29; ANA: 94.31±1.52; 3-HANA: 97.08±3.43; XA: 94.98±3.36; QUINA: 97.84±2.67; 5-HIAA 98.55±2.05. | | | | | | | |

**Table S4.** Spearman’s correlations of the serum kynurenine metabolites levels with CGI. GAF and PANSS scales scores in FES patients.

|  | **GAF**  **(ρ)** | **CGI**  **(ρ)** | **PANSS**  **total (ρ)** | **PANSS**  **positive (ρ)** | **PANSS**  **negative (ρ)** | **PANSS**  **general (ρ)** |
| --- | --- | --- | --- | --- | --- | --- |
| **Trp** | 0.07 | 0.10 | 0.07 | -0.19 | 0.05 | 0.17 |
| **KYN** | -0.05 | 0.07 | 0.18 | 0.14 | -0.07 | 0.13 |
| **KYNA** | -0.00 | 0.11 | -0.03 | 0.30 | -0.34 | -0.07 |
| **ANA** | -0.13 | -0.05 | 0.04 | -0.07 | 0.07 | 0.20 |
| **3-HANA** | -0.21 | -0.03 | 0.08 | 0.04 | 0.02 | 0.11 |
| **QUINA** | 0.21 | 0.06 | -0.08 | -0.18 | -0.25 | 0.08 |
| **5-HIAA** | -0.00 | 0.10 | 0.28 | 0.22 | -0.02 | 0.17 |
| **XA** | -0.05 | 0.16 | 0.18 | 0.11 | -0.12 | 0.29 |
| **3-HK** | 0.03 | -0.04 | -0.08 | 0.09 | -0.23 | -0.07 |
| ρ values refer to Spearman’s correlation. **FES –** first episode of schizophrenia.  For abbreviations of kynurenine metabolites see main text. | | | | | | |

**Table S5.** Spearman’s correlations of the serum kynurenine metabolites levels with CGI and GAF scales in MES patients.

|  | **GAF**  **(ρ)** | **CGI**  **(ρ)** | **PANSS**  **total (ρ)** | **PANSS**  **positive (ρ)** | **PANSS**  **negative (ρ)** | **PANSS**  **general (ρ)** |
| --- | --- | --- | --- | --- | --- | --- |
| **Trp** | -0.08 | 0.04 | -0.20 | -0.09 | -0.29^*^ | -0.10 |
| **KYN** | 0.30^*^ | -0.16 | -0.23 | -0.27^*^ | -0.17 | -0.12 |
| **KYNA** | 0.10 | -0.14 | -0.10 | -0.26^*^ | 0.00 | -0.00 |
| **ANA** | 0.09 | -0.17 | 0.02 | -0.04 | -0.06 | 0.05 |
| **3-HANA** | -0.03 | 0.20 | -0.02 | 0.02 | -0.06 | 0.03 |
| **QUINA** | 0.07 | -0.29^*^ | 0.01 | 0.11 | 0.01 | -0.07 |
| **5-HIAA** | 0.34^*^ | -0.13 | -0.23 | -0.22 | -0.22 | -0.10 |
| **XA** | 0.19 | -0.01 | -0.04 | -0.01 | -0.04 | -0.00 |
| **3-HK** | 0.02 | 0.15 | 0.16 | 0.09 | 0.08 | 0.16 |
| ρ values refer to Spearman’s correlation. *p≤0.05  **MES** – multiple episodes of schizophrenia.  For abbreviations of kynurenine metabolites see main text. | | | | | | |

**Table S6.** Neuropsychological Test Battery according to the domains of the “Measurement and Treatment Research to Improve Cognition in Schizophrenia”.

| Cognitive domain | Variables |
| --- | --- |
| **Speed of Processing**  Trail Making Test-A subtest (TM A) (nonverbal) (Reitan and Wolfson. 1985) Stroop Word Test (Stroop W): word reading (verbal) (Stroop. 1935)  Verbal Phonemic Fluency (all words starting with F. P. and L) (Novelli et al.. 1986) | 1. time in seconds 2. number of words read correctly in 30 seconds 3. sum of words produced in 60 seconds |
| **Sustained attention/vigilance**  Wisconsin Card Sorting Test (WCST) (Grant and Berg. 1948) | 4. number of non perseverative errors (NPEs) |
| **Working memory**  Corsi block test: spatial span (Orsini et al.. 1987)  Trail Making B-A subtest (TM B-A) (Reitan and Wolfson. 1985) | 1. raw score correct 2. differential score between subtests B and A (time in seconds) |
| **Verbal learning**  Buschke Verbal Selective Reminding Test (BVSRT) (Buschke. 1973) | 7. delayed recall 15 minutes after 6 learning trials |
| **Visual learning**  Rey-Osterrieth Complex Figure (ROCF) (Osterrieth. 1944) | 8. delayedrecallafter15 minutes |
| **Reasoning and problem solving**  WCST (Grant and Berg. 1948)  Raven's Coloured Progressive Matrices (RCPM) (Raven. 2008) | 1. number of completed categories (CCs) 2. number of correct answers |
| **Social cognition**  Facial Affect Recognition (FAR): photographs of emotional faces and emotion labels were presented on a computer screen. Participants were asked to choose 1 of 6 emotions explicitly specified on the monitor for given face (subtest A) or to select 1 of 6 faces that corresponded to the emotion displayed (subtest B) (Ekman and Friesen. 1971) | 11. sums of named (subtest A) and recognized (subtest B) emotions |

**Table S7**. Adjusted (covariates: age. years of education) correlations (p values) of levels of serum kynurenine metabolites with cognitive domains in a subgroup of patients affected by schizophrenia (n=43).

|  | **Speed of processing** | **Sustained Attention** | **Working Memory** | **Verbal Learning** | **Visual Learning** | **Reasoning** | **Social Cognition** |
| --- | --- | --- | --- | --- | --- | --- | --- |
| **Trp** | 0.08 | 0.26 | 0.35^*^ | -0.05 | 0.18 | 0.16 | 0.22 |
| **KYN** | -0.39^*^ | -0.35 | 0.01 | -0.20 | 0.14 | 0.03 | 0.06 |
| **KYNA** | -0.27 | -0.01 | 0.05 | -0.18 | 0.05 | 0.10 | 0.07 |
| **ANA** | -0.19 | 0.08 | -0.05 | -0.14 | 0.09 | 0.10 | 0.00 |
| **3-HANA** | -0.04 | -0.11 | -0.19 | -0.22 | -0.12 | -0.19 | -0.02 |
| **QUINA** | -0.11 | 0.11 | 0.04 | -0.13 | -0.02 | -0.10 | 0.12 |
| **5-HIAA** | -0.36^*^ | 0.01 | 0.04 | -0.19 | 0.19 | 0.08 | 0.10 |
| **XA** | 0.10 | 0.24 | 0.25 | -0.23 | -0.01 | 0.04 | -0.07 |
| **3-HK** | 0.15 | 0.19 | 0.07 | 0.05 | -0.17 | -0.41^*^ | -0.16 |
| p values refer to Partial correlation. *p≤0.05  For abbreviations of kynurenine metabolites see main text. | | | | | | | |

**Table S8.** Adjusted (covariates: age. years of education) correlations (p values) of levels of serum kynurenine metabolites with cognitive domains in a subgroup of FES patients (n=25).

| **p** | **Speed of processing** | **Sustained Attention** | **Working Memory** | **Verbal Learning** | **Visual Learning** | **Reasoning** | **Social Cognition** |
| --- | --- | --- | --- | --- | --- | --- | --- |
| **Trp** | 0.05 | 0.47^*^ | 0.43^*^ | 0.05 | 0.41^*^ | 0.39 | 0.44^*^ |
| **KYN** | -0.60^*^ | 0.06 | 0.03 | -0.16 | 0.07 | -0.14 | 0.17 |
| **KYNA** | -0.73^*^ | 0.10 | -0.08 | -0.28 | -0.11 | -0.11 | 0.08 |
| **ANA** | 0.10 | -0.07 | 0.04 | -0.12 | -0.14 | -0.22 | -0.20 |
| **3-HANA** | 0.06 | -0.56^*^ | -0.44^*^ | 0.28 | -0.23 | -0.26 | -0.19 |
| **QUINA** | -0.53^*^ | -0.14 | -0.05 | -0.08 | -0.00 | -0.18 | 0.15 |
| **5-HIAA** | -0.60^*^ | 0.01 | 0.04 | -0.14 | 0.13 | -0.12 | 0.22 |
| **XA** | -0.08 | 0.23 | 0.17 | -0.21 | -0.09 | 0.07 | 0.00 |
| **3-HK** | -0.03 | 0.08 | -0.09 | 0.29 | 0.32 | 0.07 | -0.06 |
| p values refer to Partial correlations. *p≤0.05  For abbreviation of kynurenine metabolites see main text. | | | | | | | |

**Table S9.** Adjusted (covariates: age. years of education) correlations (p values) of levels of serum kynurenine metabolites with cognitive domains in a subgroup of MES patients (n=18).

| **Speed of processing** | | **Sustained Attention** | **Working Memory** | **Verbal Learning** | **Visual Learning** | **Reasoning** | **Social Cognition** |
| --- | --- | --- | --- | --- | --- | --- | --- |
| **Trp** | 0.24 | 0.29 | 0.43 | -0.16 | -0.13 | 0.05 | -0.17 |
| **KYN** | -0.23 | 0.05 | -0.03 | -0.25 | 0.47 | 0.45 | 0.06 |
| **KYNA** | 0.06 | 0.01 | 0.21 | -0.01 | 0.36 | 0.40 | 0.20 |
| **ANA** | -0.29 | 0.26 | 0.31 | 0.18 | -0.51^*^ | 0.06 | -0.29 |
| **3-HANA** | 0.14 | 0.27 | 0.36 | -0.39 | 0.38 | 0.45 | 0.05 |
| **QUINA** | 0.21 | 0.42 | 0.161 | -0.15 | 0.00 | 0.01 | 0.16 |
| **5-HIAA** | -0.20 | 0.09 | 0.01 | -0.26 | 0.47 | 0.51^*^ | 0.09 |
| **XA** | 0.16 | 0.33 | 0.39 | -0.23 | 0.09 | 0.09 | -0.16 |
| **3-HK** | 0.25 | 0.26 | 0.28 | 0.14 | -0.27 | -0.56^*^ | -0.36 |
